# CCR5/CCL5 axis interaction promotes migratory and invasiveness of pancreatic cancer cells

**DOI:** 10.1038/s41598-018-19643-0

**Published:** 2018-01-22

**Authors:** Santosh Kumar Singh, Manoj K. Mishra, Isam-Eldin A. Eltoum, Sejong Bae, James W. Lillard, Rajesh Singh

**Affiliations:** 1 Morehouse School of Medicine, Department of Microbiology, Biochemistry and Immunology, Atlanta, GA 30310 USA; 2 Cancer Biology Research and Training Program, Department of Biological Sciences, Alabama State University, Montgomery, AL 36101 USA; 3 Department of Pathology, University of Alabama at Birmingham, Birmingham, AL 35294 USA; 4 Department of Medicine, University of Alabama at Birmingham, Birmingham, AL 35294 USA

## Abstract

Pancreatic cancer (PC) is one of the deadliest cancers and remains a major challenge due to its invasive and metastatic nature. Increased levels of CCR5 and CCL5 have established indicators for disease status in various cancers, including PC. However, their role in invasion and metastasis of PC is not known. Here we conducted immunohistochemistry of PC tissues and found elevated epithelial staining for CCR5 and CCL5 in metastatic PC tissues compared to non-neoplastic. *In vitro* experiments, such as flow cytometry, immunofluorescence and western blotting with human PC cell lines (AsPc-1, BxPc-3 and MIA PaCa-2), showed higher expression levels of CCR5. The CCL5 activation of PC cells expressing CCR5 increased their invasive potential, while treatment with CCR5 inhibitor maraviroc inhibited the CCL5 activation. CCL5 induced proliferation of PC cells was mediated through F-actin polymerization, while there was marked reduction when the cells were treated with maraviroc. The direct interaction of CCR5 with CCL5 was verified using a calcium mobilization assay. Taken together, our results demonstrate that CCR5 and CCL5 are potential markers for metastatic PC cancer, and their interaction leads to the increased PC cell invasion. Thus, blocking CCR5/CCL5 axis might prove beneficial to prevent metastasis and provide a more therapeutic strategy to control PC progression.

## Introduction

Pancreatic adenocarcinoma is one of the most deadly cancers for solid malignancies and remains a major challenge in oncology because of its poor response to chemotherapy and radiation as well as its invasive and metastatic nature^1^. As evidenced by the fact that the 5-year survival rates of pancreatic cancer (PC) patients are below 5%, the mortality rate equals its incidence^2,3^. This is because, the majority of pancreatic cancers (PCs) are diagnosed at an advanced stage, beyond any possibility of cure^4^. Current predictions suggest that PC death rates are on the rise^5^. Despite a progressive advancement in potential chemotherapeutics to cure cancer, agents effective in other cancer types were found to be unsuccessful in PC cells^3^. The most intimidating factor of PC is the lack of symptoms and its highly aggressive malignancy with invasive and metastasizing properties^2^. These features indicate that PC possesses unique mechanisms that are not yet well understood. A better understanding of the early neoplastic changes within the pancreas will help in diagnosis and prevent the progression of PC^4^. In addition to this, the second criterion that determines the fate of patients with PC is its distant metastasis that is detected in two-thirds of the patients. The most common site of distant metastasis in PC is the liver and then the brain^2,6^.

Many aspects of a series of molecules were found to implicate the progression and metastasis of cancer cells. However, the precise mechanism involved in the directional migration of cancer cells to distant organs is not clearly known^7^. Chemokines are proinflammatory chemoattractant cytokines that function primarily in leukocyte trafficking and other biological activities, such as development, angiogenesis, and hematopoiesis^8^. Chemokines bind to their cognate receptors, most of which belong to the G-protein coupled receptor family, and are expressed on endothelial cells and lymphocytes. In addition to their role in several pathological conditions, it has become progressively evident that chemokines and their receptors find a significant position in determining the metastatic destination of tumor’s cells^9^.

Among the known chemokines, CCL5 (CC chemokine ligand 5) also known as RANTES (regulated on activation, normal T cell expressed and secreted), strongly promotes carcinogenesis and stroma genesis, which was initially recognized for its important role in inflammatory diseases^10^. CCL5 has three different chemokine C-C motif receptors (CCRs): CCR1, CCR3, and CCR5^11^. CCL5 was also revealed to bind G protein-coupled receptor 75 (GPR75)^12^. CCL5 reported to be produced by cancer cells or nonmalignant stromal cells at the primary or metastatic sites^13^. Thus, the elevated level of CCL5 in tissues or plasma is indicative of unfavorable outcome in patients with either melanoma, breast, cervical, prostate, gastric or even pancreatic cancer^10,14^. Among the receptors of CCL5, its interaction with CCR5 was very well established and elucidated in tumor progression and recruitment of tumor infiltration leukocytes in several cancer types. Evaluating the mechanism of pancreatic adenocarcinoma cell evasion from the immune system highlighted the importance of CCL5/CCR5 interaction. CCR5 is expressed on various immune cell populations such as macrophages, dendritic cells and memory T cells in the immune system; endothelium, epithelium, vascular smooth muscle and fibroblasts; microglia, neurons, and astrocytes in the central nervous system^15^. In addition, its expression on cancer cells, along with CCL5 has found to play an important role in cancer progression and metastasis. It is reported that in human breast cancer, specimens increased expression of CCR5 along with its ligand CCL5 in the basal and HER-2 genetic subtypes^16^. Besides, CCL5 has gained an utmost importance as an inflammatory chemokine, CCL5 and CCR5 were regarded as a poor prognosis signature marker in various cancer types such as renal^17^, prostate^18^, breast^19^, cervical^20^, lung^21^ and ovarian^22^ cancers. However, CCR5/CCL5 participation in activating invasion and metastasis of PC has not been reported yet.

In this study, we present our investigative reports on CCR5/CCL5 expression in PC cases and show their association with disease progression using immunohistochemistry staining. We further investigated the effect of CCL5 on CCR5 expressing cells by a series of *in vitro* experiments by CCL5 stimulation and CCR5 blockade and also reported that the CCR5/CCL5 axis played a major role in PC cell invasion and metastasis.

## Results

### Pancreatic cancer tissues and cells express CCR5 and CCL5

**Figure 1:**
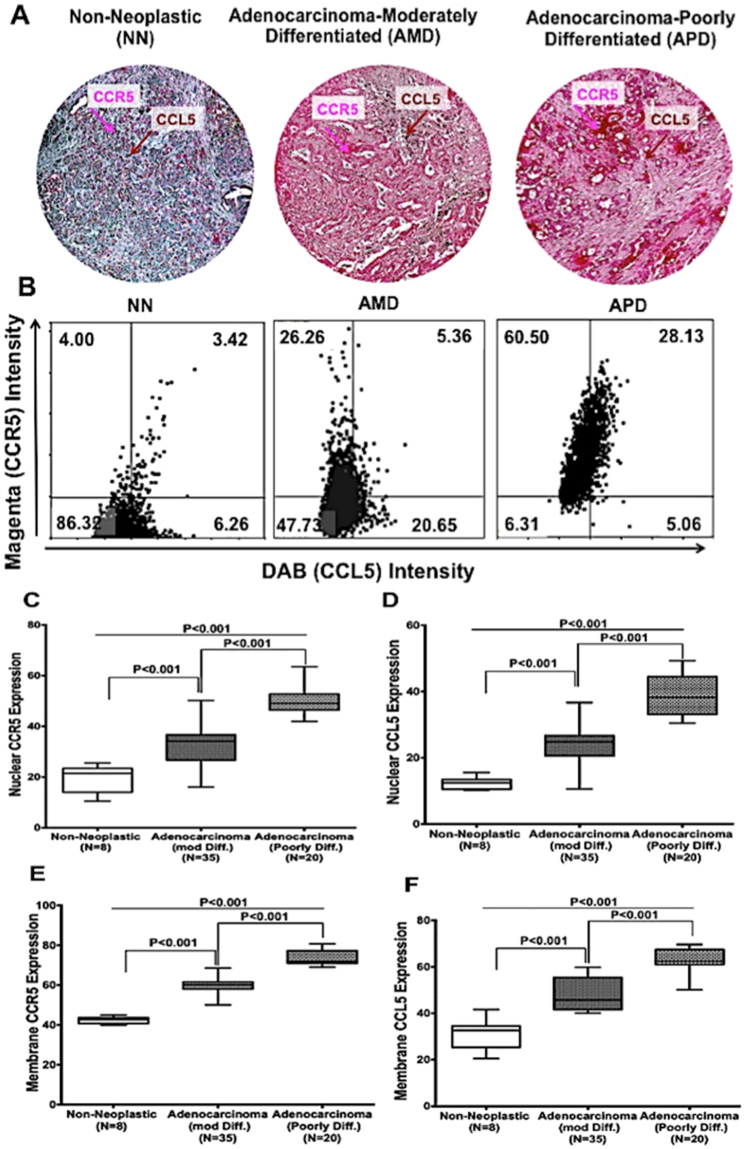
CCR5 and CCL5 expression in pancreatic cancer tissues: (**A**) Pancreatic tissues from non- neoplastic (N = 8), moderately differentiated (N = 35) and poorly differentiated (N = 20) were stained with anti-CCL5 and anti-CCR5 antibodies. Magenta (AP) color show CCR5 and brown (DAB) color show CCL5 staining. An Aperio ScanScope CS system with a 40 × objective captured digital images from the slide. Representative cases are indicated immuno-intensities of CCL5 and CCR5 using image analysis Aperio ImageScope v.6.25 software. (**B**) Scatter diagrams were used to plot stain mean intensities of Magenta (AP) color show CCR5 and brown (DAB) color show CCL5 staining using an automatic algorithm function using HistoQuests software feature. (**C–F**) Pancreatic tissues from non- neoplastic (N = 8), Moderately differentiated (N = 35) and Poorly differentiated (N = 20) were stained with anti-CCR5-CCL5 antibodies. Nuclear and membrane CCR5/CCL5 expression by pancreatic cancer tissues Brown (DAB) color shows CCL5 and magenta color show CCR5 staining. Nuclear and membrane intensity of CCR5-CCL5 were quantified using nuclear and membrane algorithm of image analysis Aperio ImageScope v.6.25 software. An Aperio Scan Scope CS system with a 40 × objective captured digital images from the slide. Stained cells were counts negative and positive membrane and categorize them as to stain intensity 0 (blue), 1 + (yellow), 2 + (orange) and 3 + (Red). There are significant differences (p < 0.001) between groups with pancreatic cancer and non-neoplastic.

It is evident from previous reports that CCR5 and CCL5 are responsible for cancer progression and metastasis. However, their function in PC remains unknown. In this regard, tissue microarrays (TMAs) of PC patients, which included 69 cases, were stained and analyzed for both CCL5 and CCR5. Representative images of PC TMAs were shown in Fig. 1A, where nuclear and membrane expression of CCR5 and CCL5 were found to be high in adenocarcinoma (moderately and poorly differentiated) compared to non-neoplastic. Among the adenocarcinoma tissues, the poorly differentiated tissues had high expression of CCR5 and CCL5.We further quantify the expression level in tissue using the Histo Quest software; hematoxylin staining was used as a master marker for cell identification on the basis of nuclear detection. The range of intensities of the Magenta and the DAB stainings were set by auto detection of the software (Fig. 1B). The results are visualized in dot plot scatter diagrams. Cut-offs (to differentiate between positive and negative cells) and gates (to accentuate between cell populations) were set in the dot blots. The poorly and moderately differentiated adenocarcinomas showed CCR5 intensity 60.5% and 26.26% compared to non –neoplastic tissues (4%). The expression of CCR5 and CCL5 in nuclear and membrane were analyzed using Aperio software (Fig. 1C–F). Both the nuclear and membrane expression of CCR5 were found to be higher in poorly differentiated adenocarcinoma than in moderately differentiated and non-neoplastic tissues (median values 50 and 70 versus 35, 60 and 21, 42). Although CCR5 expression in poorly differentiated was slightly higher than moderately differentiated. However, the nuclear and membrane expression of CCL5 were found to be similar to poorly differentiated adenocarcinoma cells of CCR5 with less average median value (37, 22, 16 for nuclear expression of CCL5 in poorly, moderately and non-neoplastic cells). For the membrane expression of CCL5, average median value was found 60, 42 and 32 respectively for poorly, moderately and non-neoplastic.

**Figure 2:**
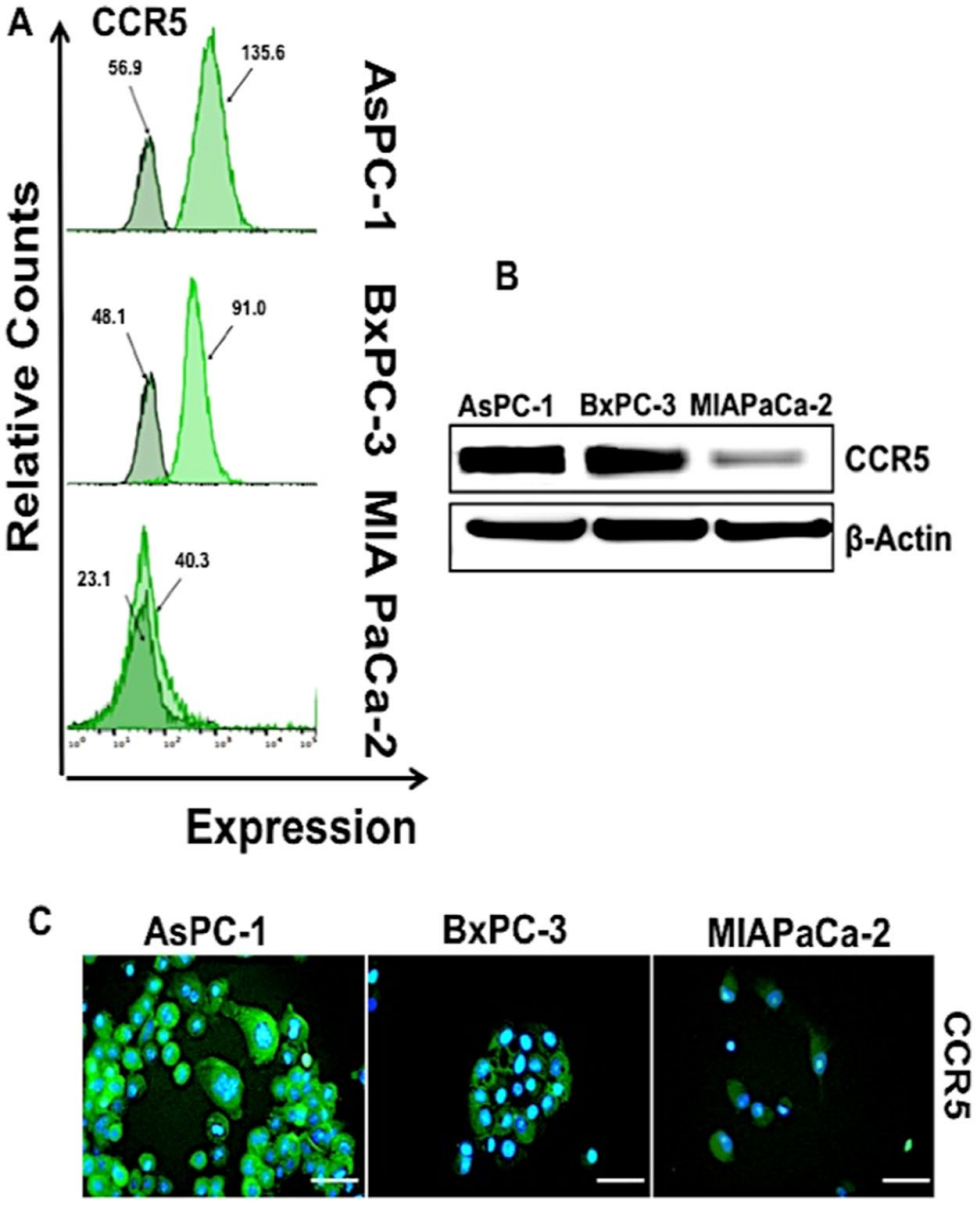
Expression of CCR5 by pancreatic cancer cells (**A**) AsPC-1; BxPC-3 and MIA PaCa-2 cells were stained with FITC-conjugated isotype or anti-CCR5 antibodies and quantified by flow cytometry. Histograms represent isotype and respective CCR5 fluorescent intensity. (**B**) Total protein (~30 µg) from AsPC-1, BxPC-3 and MIA PaCa-2 cell line was resolved by SDS PAGE gel. Expressions of CCR5 were detected by western blot analysis with anti-CCR5 antibodies. β-Actin antibody was used as a loading control for all the samples. (**C**) CCR5 expression levels in AsPC-1, BxPC-3 and MIA PaCa-2 cells, Immunostained with anti-CCR5 antibody and all the cells nuclei were counterstained with DAPI. Bars correspond to the mean ± S.D. Data are representative of three independent experiments.

Similar results were observed with PC cell lines AsPc-1, BxPc-3 and MIA PaCa-2 were analyzed using flow cytometry (Fig. 2). Among the three cell lines, AsPc-1 and BxPc-3 showed higher CCR5 expression compared to MIAPaCa-2. The mean fluorescence intensity was found to be higher in AsPC-1 (135.6) relative to BxPC-3 (91.0) and MIA PaCa-2 (40.3). The observed expression profiles of CCR5 were further corroborated with fluorescence microscopy and western blot analysis (Fig. 2B and C).

### CCR5 expression is high at mRNA level in PC cells

**Figure 3:**
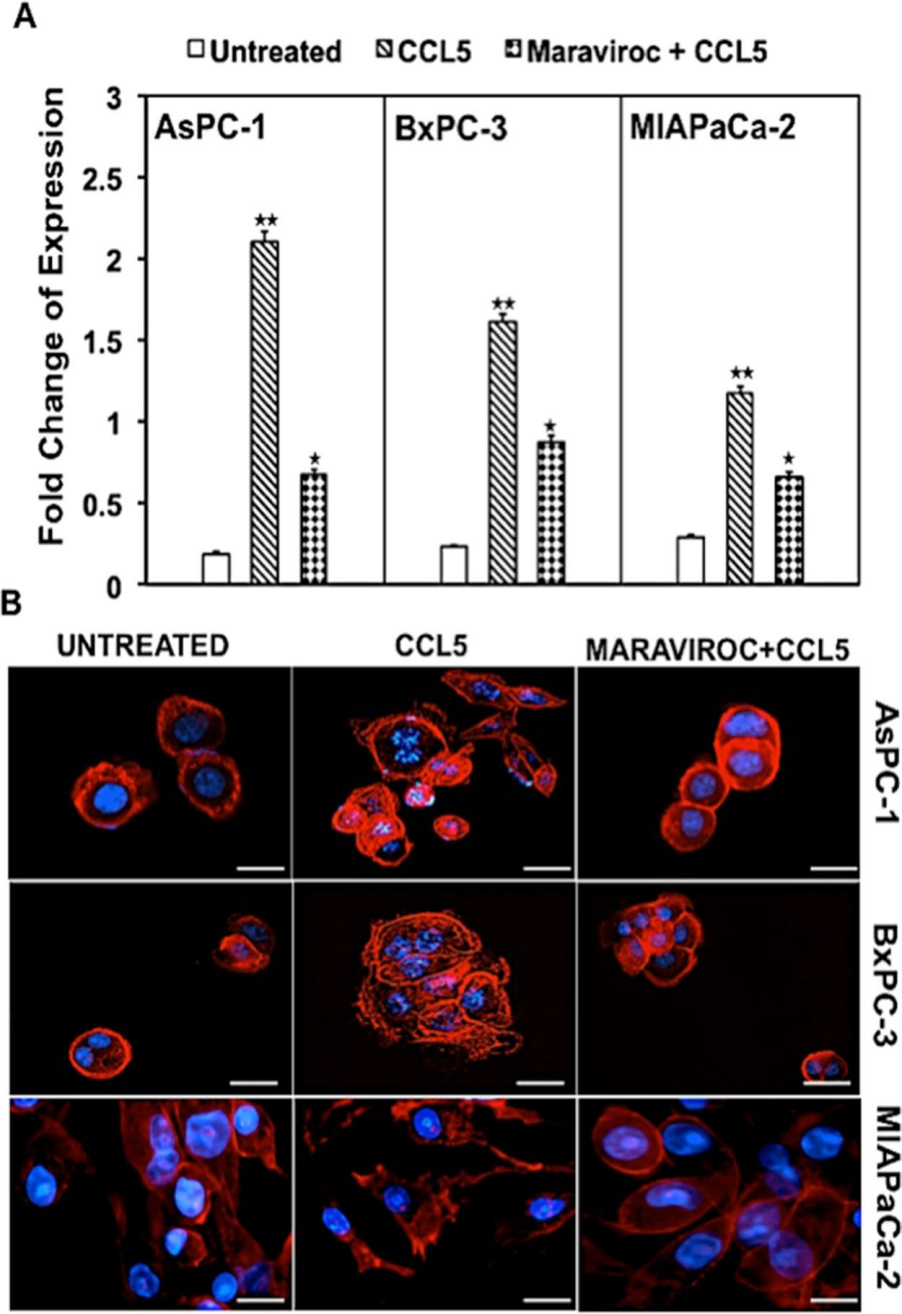
Expression of CCR5 on mRNA and F-actin polymerization of pancreatic cancer cells: (**A**) Quantitative RT-PCR analysis showed fold change expression in mRNA expression of CCR5 in AsPC-1, BxPC-3 and MIA PaCa-2 cell lines. Data were normalized to the levels of the housekeeping gene 18 S expression, and the experiments were repeated three times. Data are presented as fold change in expression ( ± standard error) and the asterisks indicate significance determines by student *t*-test (*P < 0.05; **P < 0.01). (**B**) AsPC-1, BxPC-3 and MIA PaCa-2 were induced by 100 ng/ml CCL5 and pseudopodium formation were observed by fluorescence microscope. However, morphological changes in actin filament were not observed in untreated cells and CCR5 antagonist maraviroc.

To further characterize the chemokine-receptor expression and identify the function of CCL5 in PC, pancreatic cancer cell lines were analyzed through RT-PCR using our optimized primer sets for CCR5. The CCR5 expression at mRNA levels in presence of CCL5 as shown in Fig. 3A increased 11, 7 and 4 fold respectively in AsPC-1, BxPC3, and MIA PaCa-2 cell lines as compared to untreated and decreased by 3, 1.9 and 1.8 fold after the inhibitor maraviroc treatment in all the three cell lines. Similar to the protein levels, the mRNA expression was higher in AsPC-1 > BxPC3 > MIA PaCa-2. These observations indicate a chemokine-receptor interaction based regulation of the chemokine receptor expression at the transcriptional level.

### CCR5/CCL5 axis induces F-actin polymerization in PC

Several factors were found to be responsible for the invasion of cancer cells among the cytoskeleton organization that plays an important role and actin filaments are leading edge for the cell migration. In this study, we sought to determine the F-actin cytoskeleton morphological changes using immunofluorescence during the CCR5/CCL5 interaction. As shown in Fig. 3B, stimulation of all the three cell lines with CCL5 increased the actin stress fibers compared to the control that was left untreated. To confirm that CCL5 is the principal agent involved in the expression of action, adding maraviroc in the medium along with CCL5 blocks the CCL5 activity. The cells treated with inhibitor showed a decreased actin expression and the cells resembled as that of control cells.

### CCR5/CCL5 interaction is functional in PC (Ca mobilization assay)

**Figure 4:**
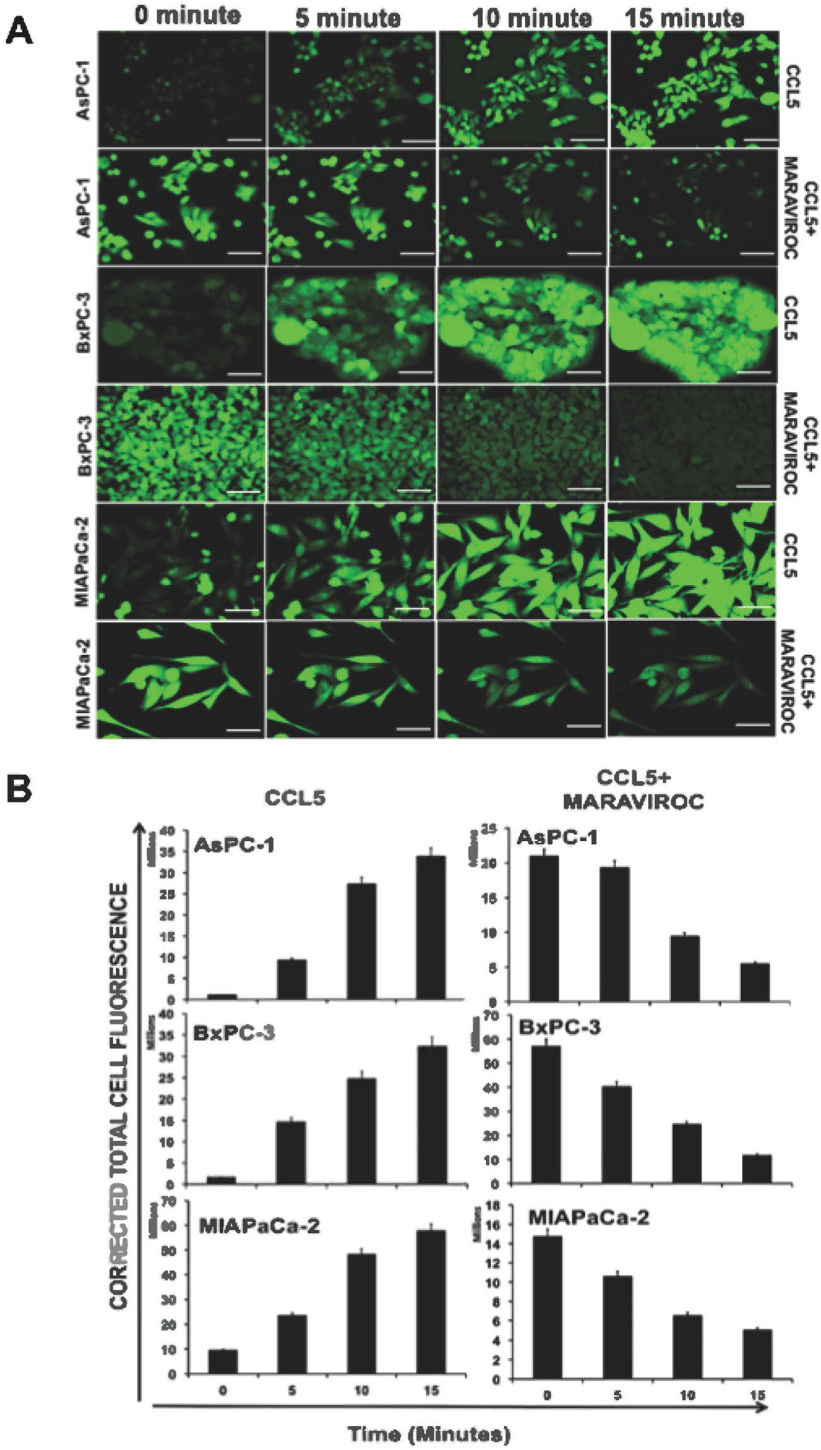
Calcium mobilization assay in pancreatic cancer cells. (**A**) Immunofluorescence staining was performed for the cells treated with CCL5 200 ng/ml and CCR5 antagonist maraviroc for different 5, 10, and 15 minute time point and the images were captured by fluorescence microscopy. (**B**) Total cell fluorescence intensity in AsPC-1, BxPC-3 and MIA PaCa2 are plotted in a bar graph. Addition of CCL5 induced maximum expression of CCR5 at the 15-minute time. However, the addition of inhibitor reversed the intensity of calcium mobilization in cells.

Calcium mobilization assay utilizes a calcium-sensitive fluorescent dye that binds to calcium released into a cytoplasm in response to ligand activation. The assay measures the G-protein coupled receptor activation or inhibition based on intracellular fluorescence intensity. Based on the above findings in PC cells, Ca^+^ mobilization studies were performed to determine that the CCR5 expressed on the surface of the PC cells and its interaction with CCL5 was functional. In this study, the PC cells were labeled with FluroForte AM before the cells were challenged with ligand (CCL5). The effect of CCL5 and maraviroc on the calcium induction is shown in Fig. 4A and the intensity of total cell fluorescence are presented in Fig. 4B. The addition of CCL5 induced the calcium concentration in a time-dependent manner in all three cell lines (AsPc-1, BxPc-3, MIA PaCa-2) irrespective of differential expression of CCR5 as observed at protein levels. The functional interaction of CCR5/CCL5 was further determined by using CCR5 inhibitor maraviroc. The addition of inhibitor reversed the intensity of calcium mobilization in cells indicating that CCL5 indeed interacts with CCR5.

### Blocking of CCR5/CCL5 axis abrogates migration and invasion of PC cells

**Figure 5:**
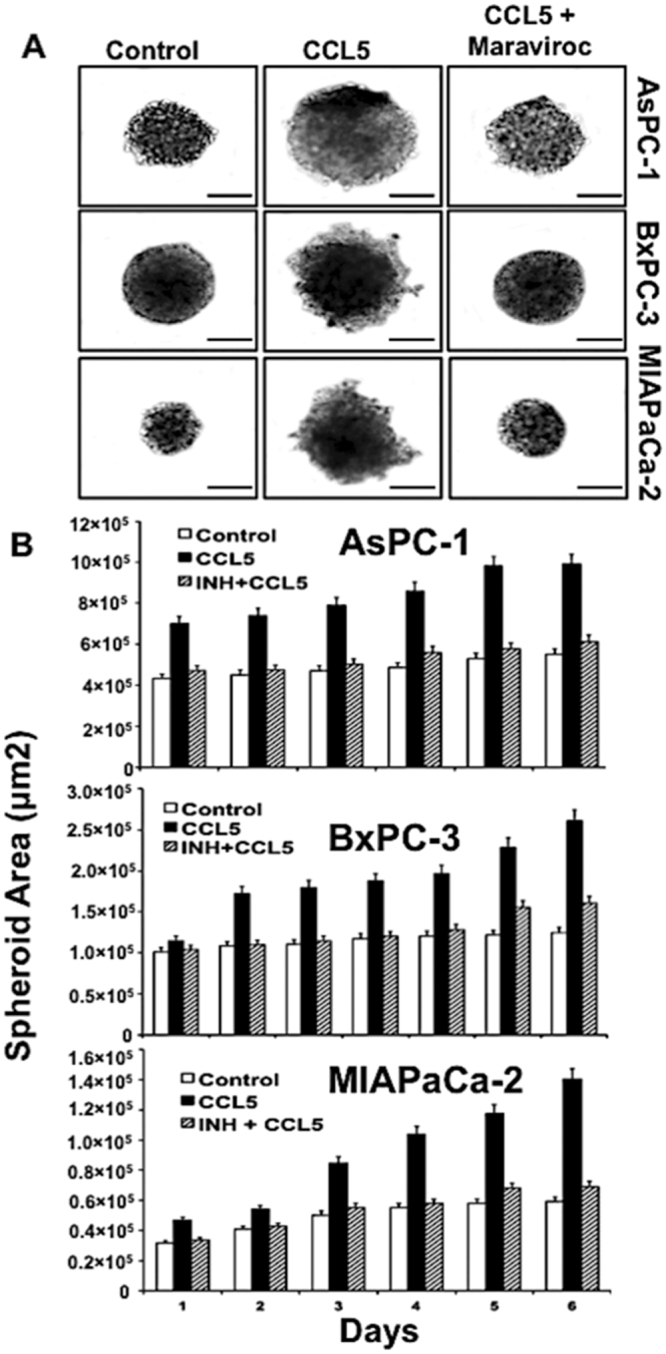
Migration and invasion assay in pancreatic cancer cells. (**A**) Morphology of 3D cell invasion over a 6-day period. With the addition of CCL5 as a chemotactic attractant in the medium, the invading cells projected out of the spheroid into the matrix, however, non-invading cell remain as aggregates and do not invade into the surrounding matrix. The inhibition of CCL5 was performed by maraviroc confirm the role of CCL5/CCR5 interaction mediated invasion of PC cells. (**B**) Quantitative analysis of surface area for pancreatic cancer cells (AsPC-1, BxPC-3 and MIA PaCa2) over a period of 6 days.

A significant feature of PC is its metastatic behavior that invades other organs. In order to determine the functional significance of CCR5/CCL5 interaction leading to the invasion of PC cell, we used the spheroid cell invasion assay using all the three PC cell lines (AsPc-1, BxPc-3, MIA PaCa-2), which displayed a differential expression of CCR5 and CCL5. With the addition of CCL5 as a chemotactic attractant in the medium, the invading cells projected out of the spheroid into the surrounding matrix with spindle-like projections. However, such invasion was not observed in the control cells where the chemokine was not added (Fig. 5A and B). In order to substantiate that the invasion was due to the chemotactic attraction of CCR5 with CCL5, we inhibited the function of CCL5 using maraviroc. In this test, the invasive capability of the PC cells was abrogated, and they resembled that of the control cells. These findings indicated the potential role of CCL5/CCR5 interaction mediated invasion of PC cells.

## Discussion

Cancer has been considerably emphasized as an inflammatory process that creates a dynamic microenvironment^19^. The tumor microenvironment is comprised of malignant, non-transformed cells and the proteins they produce^23^. Proteins derived from non-malignant cells support the growth of cancer cells and may even promote metastasis^23^. Studies carried out in the last decade have clearly proved the concurrent concept of proteins, such as chemokines promote the autocrine growth, immune evasion and enhance invasion and migratory potential of cancer cells^9^.

Having established the importance of CCR5/CCL5 signaling axis in various cancers, their role in metastasis of pancreatic adenocarcinoma remains unknown. Previous study has shown that CCL5 was highly expressed in PC cells, however, in normal epithelial-like HPNE cells, was found minimal expression^24^. To the importance of chemokine study in tumor cells, we further extend CCR5/CCL5 interaction in selected PC cell lines. In this study, we attempted to characterize the expression and function of this classical inflammatory mediator CCL5 and its cognate receptor CCR5 in inducing invasion of PC. Interestingly, in TMA, we found that CCR5 and CCL5 were both expressed at high levels in PC that are moderately and poorly differentiated, and which were in contrast when similar characterization was made in non-neoplastic PC tissues that expressed CCR5 and CCL5 in low levels. These results suggest that CCR5 and CCL5 are preferentially expressed in high amounts during the development of specific cancer types such as metastatic^16^. This notion was supported by the fact that invasiveness of human breast cancer cells MCF-10A transduced with oncogenes; *Neu-T, H-RAS or c-SRC* increased in response to CCL5^16^. These reports suggest that oncogenic events, which promote aggressive phenotypes, activate CCL5/CCR5 expression in cancer cells^16^.

In recent years, chemokines and their receptors have gained much attention because of their significant roles in advance stages of cancer. In general, metastasizing tumor cells expressing higher levels of receptors are attracted to the chemokines and migrate to distant secondary organs or inflammation sites producing chemokine^25^. On the other hand, chemokine and receptors are produced by the same cancer cells, that may have a differential influence on the invasion and metastatic properties of cancer cells^19^. Specific examples include CCL5 and CCL25 and their cognate receptors CCR5 and CCR9 in breast and ovarian cancers, respectively^16,19,26^. Although expression of chemokine receptor on cancer cells has significance in metastasis^27^, tumor-produced chemokines had discrete roles in the biology of primary and metastatic disease, which includes (a) tumor infiltration of leukocytes, (b) regulation of antitumor immune response, (c) controlling tumor angiogenesis, (d) functioning as autocrine or paracrine growth and survival factors, and (e) invasion and migration of cells^28^. Although we could not define the reasons for the higher expression of CCL5 in our PC tissues, a functional role for CCL5 in breast cancer has been supported by previous studies to highlight a good correlation of high CCL5 expression and poor prognosis^29,30^. Previous studies have delineated the mechanisms for the over-expression of CCL5 in cancer^31^. *In vivo* studies conducted in mouse model linked to CCL5 gene promoter showed that NF-kB is critical for CCL5 expression^32^. In addition, tumor necrosis factor-alpha (TNF-α) was found associated with higher expression levels of CCL5^33^. These data support our work that the CCL5 expression observed in PC tissues is critical and an indicator of the highly adverse state of PC.

Although our results support that CCR5/CCL5 interaction in PC promotes progression and invasiveness, there are reports that contradict our findings. Work carried out by Manes and others^28^ showed a negative correlation between the CCR5/CCL5 interaction and the progression of breast cancer cells. Blockade of CCR5 enhanced the growth of the tumor xenografts bearing wild-type p53^28^. Thus, it was important for us to clarify whether CCR5/CCL5 interaction fosters the progression and metastasis of PC through increased invasion. Inhibition of CCR5 receptor has elicited significant attention in therapeutic application in a strategic control of HIV gp120 protein binding to the CCR5 expression immune cells^34,35^. The importance of CCR5 in HIV had led to the development of several inhibitors that target this receptor^35^. By using the CCR5 inhibitor maraviroc, we show here that CCL5 activated invasion is due to its interaction with CCR5. Our results are also supported by previous studies where CCR5 antagonist maraviroc could abrogate the CCR5/CCL5 mediated metastasis of breast cancer cells^36^.

Cell migration, an integrated multistep process initiated by the progression of membrane protrusion can be triggered by chemo-attractants^37^. The binding of a chemokine to their cognate G-protein coupled receptors (GPCRs) activates a series of intracellular signaling pathways that regulate the reorganization of actin cytoskeleton^38^. The consequences of this interaction led to the migration of cancer cells due to polarization, accumulation of small GTPases, Rac, Cdc42, and PI3K at the leading edge, actin polymerization and F-actin formation^39^. In other instances, CCL5/CCR5 operate via PI3K/Akt, MEK, ERK, which in turn activates NF-kB, resulting in the activations of αvβ3 integrin and contributing to cell migration^40–43^. In the present study, to verify whether the interaction of CCR5 and its ligand was functional, we detected the cytoplasmic free Ca2+ concentration and the reorganization of actin cytoskeleton after stimulation with CCL5. Although we did not characterize the protein expressions of various pathways involved in GPCRs signaling, we found that CCR5/CCL5 interaction led to the increasing polymerization of F-actin as well as Ca^2+^ elevation after stimulation by CCL5 in PC cells, which was absent in control as well as the CCL5 inhibitor, treated cells. In tumor cells, high levels of actin polymerization are needed for the formation of pseudopodia, which is the foundation and early event of efficient migration and invasion of tumor cells^44^. In the present study, we observed that PC cells treated with CCL5, marked actin polymerization, and pseudopodium formation was noted in the periphery of cells. These results indicate that the CCR5 expressed in PC cells was the functional receptor that responds to CCL5. Take together; the motility of PC cells may be associated with the cytoplasmic free Ca^2+^ concentration and the reorganization of actin cytoskeleton induced by CCL5/CCR5 axis activation.

Viewed altogether, the data indicate that CCR5 and CCL5 are expressed in PC tissues in poorly and moderately differentiated tissues show that they are the indicators of metastatic stages of PC. Through our work on PC cell lines, we show that CCL5 invokes increases in cell invasion. Certainly, our results align with the published reports that CCL5 activating CCR5 to invoke a cascade of signaling pathways that increase the capacity of PC cells to invade/migrate to other organs. In addition to the detection of cell-surface CCR5, expression of endogenous CCL5 on PC cells may indicate their major role in evading immunity and further proliferation. Our data suggest that CCR5 antagonists may be used to reduce the risk of metastasis in patients bearing PC. Our current studies are directed towards analyzing the different contributions of CCL5/CCR5 interactions in the PC microenvironment, in the context of understanding how other major pathways are influenced in metastasis and progression of PC.

## Materials and Methods

### Cell line and culture conditions

The human pancreatic cancer cell lines AsPC-1 (ATCC-CRL-1682), BxPC-3 (ATCC-CRL-1687) were cultured in RPMI-1640 with 10% Fetal Bovine serum (FBS). MIA PaCa-2 (ATCC-1420) was grown in DMEM containing 10% FBS and 2.5% horse serum supplemented with 100 µg/ml each of streptomycin and penicillin, nonessential amino acids, HEPES, 2mM L-Glutamine and 0.025 µg/ml Amphotericin-B (Fisher Scientific, Pittsburg, PA). All the cell lines were maintained in a humidified incubator containing 5%CO_2_ at 37 °C.

### Immunohistochemistry

High-density tissue microarrays (TMA) slides [A207 (V)] were procured from AccuMax Array Inc (ISU Abxis Co.). TMA slides containing clinical samples of 69 cases were diagnosed with n = 8 (non-neoplastic), n = 35 (moderately) and n = 20 (poorly) differentiated. Paraffin-embedded tissue sections were deparaffinized in xylene and rehydrated in a graded alcohol series (100%, 95%, and 70%, 5 min each). Endogenous peroxidase was blocked by 3% H_2_O_2_. Slides were rinsed with PBST (PBS + 0.05% Tween 20) and tissue sections were blocked with normal donkey serum (NDS, 5%) for 1 h at room temperature and then incubated with goat anti-human CCL5 (5 μg/ml, R & D systems, USA) antibody at 4 °C overnight. and mouse anti-human CCR5 (8 μg/ml, R & D systems, USA) for three (3) hours at room temperature. After washing (1 × PBS, three times, 15 min each), tissue sections were incubated with donkey anti-goat (Jackson Immunoresearch, USA) and/or donkey anti-mouse antibody (Jackson Immunoresearch) for CCL5 and CCR5 respectively at room temperature for 1 h. After washing with PBST (Three- times, 15 mins), tissue sections were incubated either with the Streptavidin-horseradish peroxidase (HRP, Biolegend) (CCL5) or streptavidin-alkaline phosphatase (Jackson Immuno research) (CCR5) and developed in DAB and/or alkaline phosphatase red chromogen coloring Agent. The slides were counter stained with hematoxylin and dehydrated and mounted. Digital images were captured and analyzed using an AperioScanScope scanning system (Aperio Technologies, USA). Negative controls were generated by the omission of the primary antibody. The staining intensity was scored on a scale of 5 as follows: negative (−), negligible (±), weak (+), moderate (++) and strong (+++). For Tissue FAXS, we used technology-platform of TissueGnostics GmbH (Vienna, Austria) which provides tools to quantify protein expression levels on immunohistochemically (HistoQuest) stained tissue slides. The results are visualized in dot plot diagrams. Cut-offs (to differentiate between positive and negative cells) and gates (to accentuate between cell populations) were set in the dot blots.

### Flow cytometry

AsPC-1, BxPC-3, and MIA PaCa-2 cells were grown overnight, trypsinized (0.25% trypsin), harvested and washed twice with phosphate-buffered saline (PBS) supplemented with 2% Fetal Bovine Serum (FBS) (Fisher Scientific, Pittsburgh, PA) and counted using hemocytometer (Countess II FL, Life Technology). Cells were then treated with 1 µg of Fc Block (BD Bioscience, CA) per 100 thousand cells for 15 minutes in ice. For the membrane staining, cell was incubated with FITC conjugated anti-human CCR5 (5 µL/100 µL) antibody and FITC conjugated mouse IgG2a 4 µL (Biolegend, San Diego, CA) isotype control antibodies per 100 µL FACS buffer for 30 minutes. Finally, cells were washed with FACS buffer and resuspend in 300 µL FACS buffer. Fifty thousand cells were analyzed by flow cytometer using guava easyCyte HT (EMD Millipore, Billarica, MA).

### Immunoprecipitation and Immunoblotting

Immunoprecipitation was performed using the Pierce classic Immunoprecipitation (IP) kit (Thermo Scientific, USA) and the protocol was followed as per the manufacturer’s instructions. Briefly, whole-cell lysates were prepared from monolayer cultures of pancreatic cells grown in culture plates washed with ice-cold phosphate-buffered saline and lysed with ice-cold lysis buffer. Immunoprecipitation using 5 μg rabbit polyclonal anti-human CCR5 (Life technology, USA) was performed. The immune complexes were prepared by incubating with protein A/G Agarose, washed and eluted. The concentration of the proteins was determined using bicinchoninic acid (BCA) protein assay kit (Thermo Scientific, Rockford, IL). An equal amount of proteins (30 μg) from cell lysates were denatured at boiling temperature in Laemmli buffer for 5 minutes. The resulting proteins samples were resolved in 12% sodium dodecyl sulfate polyacrylamide gel electrophoresis (SDS-PAGE) and transferred electophoretically to nitrocellulose membrane using iBlot dry blotting (Thermo Scientific Rockford, IL). The membranes containing the transferred proteins were blocked for one (1) hour at room temperature (RT) using TBS (20 mM TRIS-HCL pH 7.6, 150 mMNaCl) Fischer Scientific, Pittsburg, PA) containing 0.1% Tween 20 and 5% non-fat dry milk (Biorad, USA). Primary antibody against CCR5 was added to the membranes (diluted 1:500) and incubated overnight at 4 °C in 5% non-fat milk containing TBST (Tris-Buffered Saline-Tween 20). Membranes were washed thrice, and corresponding secondary antibodies conjugated with horseradish peroxidase (HRP) were added to the membranes and incubated for two (2) hours at RT. The membranes were washed and the immunoreactive proteins were visualized on Image Quant LAS4000 (GE Healthcare-Biosciences, Pittsburgh, PA) using chemiluminescent detection reagent (Thermo fisher Scientific, Rockford, IL). The membranes were stripped, blocked and reprobed with β-actin to ensure equal loading. The band intensities were quantified using the Image-J software (NIH).

### Immunofluorescence

Cells were seeded in sterile 24 well plates for 24 h at 37 °C in a humid atmosphere of 5% CO_2_. Growth medium was removed, and the cells were washed twice with PBS and stained with fluorochrome-conjugated CCR5 5 ul/100 uL (Biolegend, San Diego, CA) antibody for 1 h. Cells are washed thoroughly with PBS to remove the unbound antibody. All the cells nuclei were counterstained with DAPI (Blue) (Invitrogen, USA) 300 nM for 5 minutes.

To determine the F-actin cytoskeleton, cells were treated with CCL5 (100 ng/mL) (Biolegend, San Diego, CA) recombinant protein for 30 minutes. Cells were fixed with 4% paraformaldehyde (PFA) for 10 minutes and then permeabilized by saponin (0.05%) for 10 minutes. The inhibitor maraviroc (200 ng/mL) (Sigma, USA) was added to stop the activity of CCL5 in each well of the three cell lines. Then, the cells were stained with Phalloidin^TM^ Red 594 solution (1:50) (Biolegand, USA) for 25 minutes at room temperature. The nuclei were then counterstained with DAPI. Images were captured by a fluorescent microscope with the appropriate filter FITC/GFP, Texas red and DAPI for CCR5, phalloidin, and DAPI respectively using EVOS FL microscope (Thermo Scientific, USA).

### RNA isolation and Quantitative reverse transcription polymerase chain reaction (qRT-PCR)

The cells were grown, harvested and lysed with Trizol reagent (Invitrogen, Paisley, UK) followed by our previous publication for RNA extraction^45^. Briefly, cDNA was synthesized using 1 µg of RNA per 20 µl reaction mixture. Reverse transcription super mix for RT-qPCR reagent (Biorad, USA) and PCR condition were chosen following Bio-Rad protocol. Primer sequences for chemokine CCR5 and 18S were synthesized from National Center for Biotechnology Information (NCBI) gene bank database. The following sequences of the primer were used; CCR5 sense primer, 5′-GCAAGGAG ACCACCAACAG-3′, and anti-sense primer, 5′-CCCTCACTTCCAACCCAAATC-3′. RT-PCR was performed using SYBR® Green PCR master mix reagents (Biorad, USA) and gene expression was analyzed by CFX-manager software (CFX96 Real-Time System; Bio-Rad), 18 S primer (5′-GGCCCTGTAATTGGAATGAGTC-3′and 5′-CCAAGATCCAACTACGAGCTT-3′) were used as endogenous control, and the experiments were repeated three times.

### Calcium mobilization microscopy assay

Calcium mobilization microscopy assay (CMMA) was performed using the FLUOFORTE® Calcium assay kit (Enzo Life Sciences, NY, USA) and the protocol was followed as per the manufacturer instructions. Briefly, adherent cells were prepared a day before the experiment by plating the cells in the using growth medium in 5 × 10^4^ cells per well at a plating volume of 100 µL per well in 96- well plates. From the adhered cells, the growth medium was removed and FLUOFORTE® Dye-Loading Solution was added to each well and incubated for 1 hour at RT. Initially, CCL5 (100 ng/mL) (Biolegand, USA) were added to each well and the calcium efflux was monitored at an interval of 5, 10 and 15 minutes. After that inhibitor maraviroc (200 uM/mL) (Sigma, USA) was added to each well for the same time points. A fluorescent microscope captured images with the appropriate filter FITC/GFP using EVOS FL microscope (Thermo Scientific, USA). The total corrected cellular fluorescence (TCCF) intensities were calculated using the formula^46^ (TCCF = integrated density- area of selected cell × mean fluorescence of background reading) and the fluorescent measurement was performed with Image-J software (NIH).

### Migration and Invasion assay

Migration and Invasion were performed using Cultrex96 well 3D Spheroid BME Cell Invasion Assay kit (Amsbio, MA, USA). For the migration assay; all the three cell lines (AsPC-1, BxPC-3, MIA PaCA-2) harvested, count (5 × 10^3^ cells), and seeded in 3D culture qualified 96 well spheroid formation plate and placed in culture incubator for 72 hrs at 37 °C to promote spheroid formation. After 72 hrs, cells were incubated with invasion matrix to each well of the 3D culture plate and treated with CCL5 and the combination of CCL5 and inhibitor maraviroc followed by manufacturer protocol. Cells containing invasion-modulating compound were incubated for six (6) days at 37 °C. Images were captured in each well every 24 hrs using 10 × objectives of the microscope. The changes in the area of the invasive structures were analyzed using the Image-J software (NIH).

### Statistical Analysis

CCR5 and CCL5 expression intensity in TMAs were tested for normality assumptions using the Shapiro-Wilk test and transformed to a logit scale. The general linear models (GLM) procedure was used to test the association of CCR5 and CCL5 expression and disease condition using SAS version 9.1.3 statistical analysis software. Results were declared significant at a α level of 0.05. The experimental data were compared using a two-tailed Student’s t-test and expressed as the mean ± SEM. The results were analyzed using the Stat view II program (Abacus Concepts, Inc.) and were labeled statistically significant if p values < 0.05. Using the flow Jo Software, the Kolmogorov-Smirnov (K-S) two-sample test was used to calculate the statistical significance of the CCR5 histograms.

### Data Availability

All data generated during this study are included in this published article.

## Electronic supplementary material

Expression of CCR5 by pancreatic cancer cells
