# Supplementary material for: CCR5/CCL5 axis interaction promotes migratory and invasiveness of pancreatic cancer cells
Source: Sci Rep. 2018 Jan 22;8:1323. doi: 10.1038/s41598-018-19643-0 (PMC5778036; doi:10.1038/s41598-018-19643-0)

CCR5 /CCL5 axis interaction promotes migratory and invasiveness of pancreatic cancer cells

Santosh Kumar Singh[1], Manoj K. Mishra[2], Isam-Eldin A. Eltoum[3], Sejong Bae[4], James W. Lillard Jr.[1], and Rajesh Singh[1, *]

[1]Morehouse School of Medicine, Department of Microbiology, Biochemistry and Immunology, Atlanta, GA, 30310, USA

[2]Cancer Biology Research and Training Program, Department of Biological Sciences, Alabama State University, Montgomery, AL, 36101, USA

[3]Department of Pathology, University of Alabama at Birmingham, Birmingham, AL, 35294, USA

[4]Department of Medicine, University of Alabama at Birmingham, Birmingham, AL, 35294, USA

Send correspondence to: *Rajesh Singh, Morehouse School of Medicine, Department of Microbiology, Biochemistry and Immunology, 720 Westview Drive SW, Atlanta GA 30310, Tel: 404-756-6661, Fax: 404-752-1179, E-mail: rsingh@msm.edu

Supplementary Figure 2B

Supplementary Figure 2B: **Expression of CCR5 by pancreatic cancer cells** (A) Western blot expression of CCR5 by pancreatic cancer cells (AsPC-1, BxPC-3 and MIA PaCa-2) was resolved by the SDS-PAGE gel. (B) Expression of β-actin was detected by western blot analysis with anti- β-actin antibodies. β-actin was used as a loading control for all the three samples.

CCR5 Expression

(A)

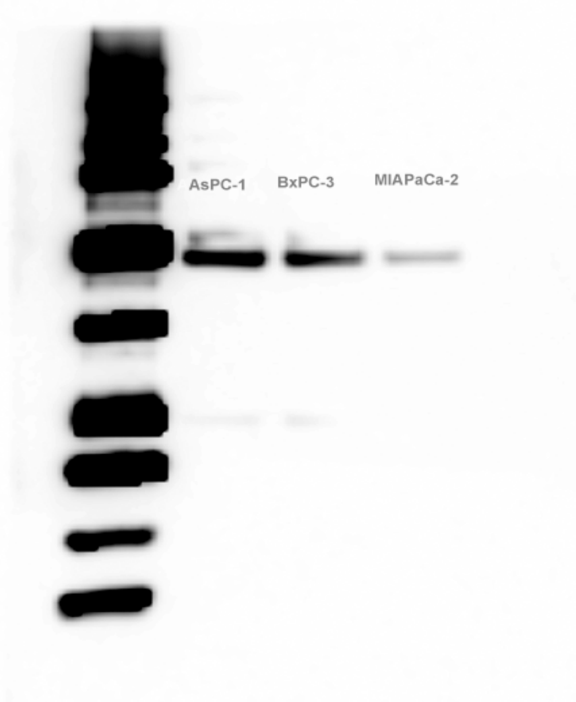

(B) **β-Actin Expression**

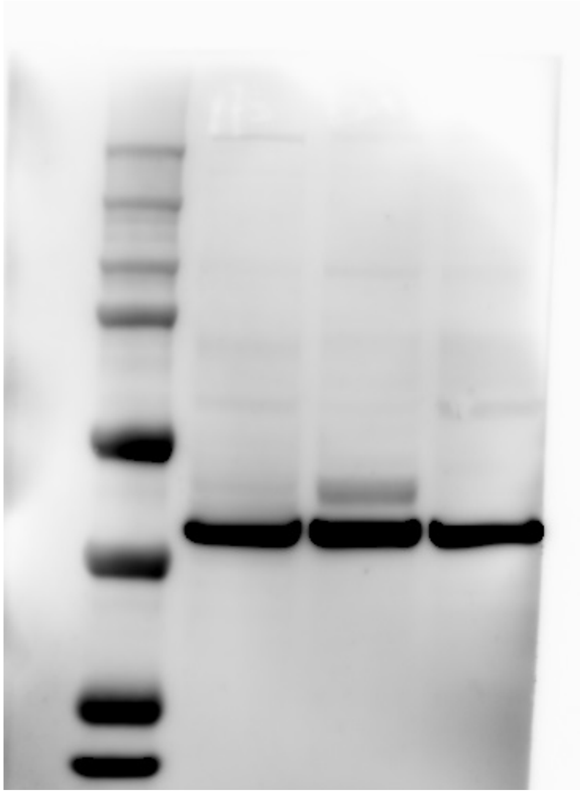

Supplement: Supplementary file 1 — Expression of CCR5 by pancreatic cancer cells [file 41598_2018_19643_MOESM1_ESM.pdf]
